# Supplementary material for: Cavity-mediated exciton hopping in a dielectrically engineered polariton system
Source: Nat Commun. 2026 Apr 24;17:3779. doi: 10.1038/s41467-026-72043-1 (PMC13106776; doi:10.1038/s41467-026-72043-1)
Supplement: Supplementary file 1 — Supplementary Information [file 41467_2026_72043_MOESM1_ESM.pdf]

Supplementary Information: Cavity-mediated exciton hopping in a dielectrically engineered polariton system

Lukas Husel,[1] Farsane Tabataba-Vakili,[1,2,3] Johannes Scherzer,[1] Lukas Krelle,[1,4] Ismail Bilgin,[1] Samarth Vadia,[1] Kenji Watanabe,[5] Takashi Taniguchi,[6] Iacopo Carusotto,[7] and Alexander Högele[1,2]

[1]*Fakultät für Physik, Munich Quantum Center, and Center for NanoScience (CeNS), Ludwig-Maximilians-Universität München, Geschwister-Scholl-Platz 1, D-80539 München, Germany*

[2]*Munich Center for Quantum Science and Technology (MCQST), Schellingstr. 4, D-80799 München, Germany*

[3]*Institute of Condensed Matter Physics, Technische Universität Braunschweig, 38106 Braunschweig, Germany*

[4]*Present affiliation: Institute for Condensed Matter Physics, TU Darmstadt, Hochschulstr. 6-8, D-64289 Darmstadt, Germany*

[5]*Research Center for Electronic and Optical Materials, National Institute for Materials Science, 1-1 Namiki, Tsukuba 305-0044, Japan*

[6]*Research Center for Materials Nanoarchitectonics, National Institute for Materials Science, 1-1 Namiki, Tsukuba 305-0044, Japan*

[7]*Pitaevskii BEC Center, INO-CNR and Dipartimento di Fisica, Universita di Trento, via Sommarive 14, I-38123 Trento, Italy*

SUPPLEMENTARY NOTE I: CONFOCAL PHOTOLUMINESCENCE SPECTROSCOPY

To investigate the effect of the engineered dielectric environment on the exciton resonance energies, we performed confocal cryogenic photoluminescence (PL) spectroscopy on the fabricated device. For this measurement, the planar cavity mirror with the van der Waals heterostack on top was mounted in backscattering geometry inside a closed-cycle cryostat held at 4.3 K. The CW pump laser had a wavelength of 675 nm, the spatial resolution in the transverse direction was approx. 1 μm.

A raster-scan map of integrated PL for the representative etch site area P_2 is shown in Supplementary Fig. 1a. In Supplementary Fig. 1b, we plot a spectrally resolved PL linecut across the etch site pair, measured along the dashed line in Supplementary Fig. 1a. The PL spectra are dominated by the resonance of excitons in the encapsulated monolayer region, and exhibit a redshifted shoulder at the position of the etch sites, with energetic positions indicated by white dots. This additional resonance originates from excitons localized to the through-hole area, with exciton binding energy and TMD band gap modified by the local

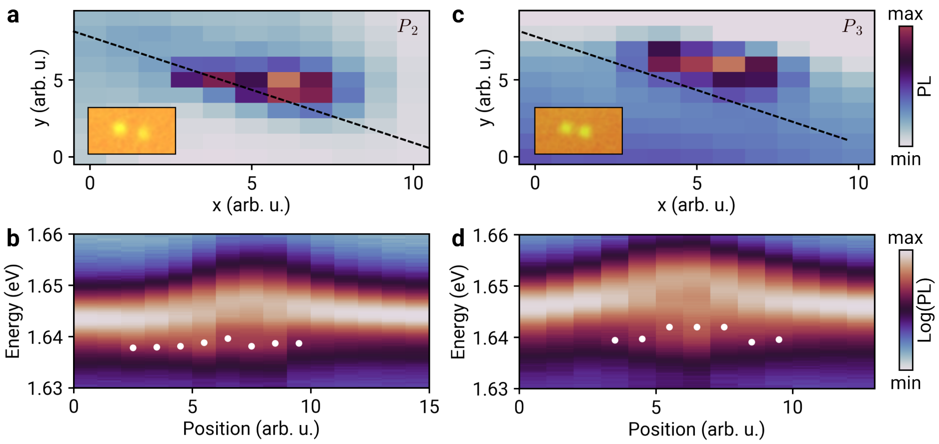

SUPPLEMENTARY FIG. 1. **Confocal photoluminescence spectroscopy.** **a**, Raster-scan map of etch site pair P_2 in cryogenic confocal PL, integrated in the spectral range 1.61 to 1.72 eV. The inset shows a microscope image of the investigated device area. **b**, Spectrally resolved PL-linecut along the dashed line in **a**. The white dots indicate the energy of a redshifted shoulder, obtained from the fit of two Gaussians to the PL spectra at each position. **c** and **d**, Same as **a** and **b**, respectively, but for the etch site pair P_3.

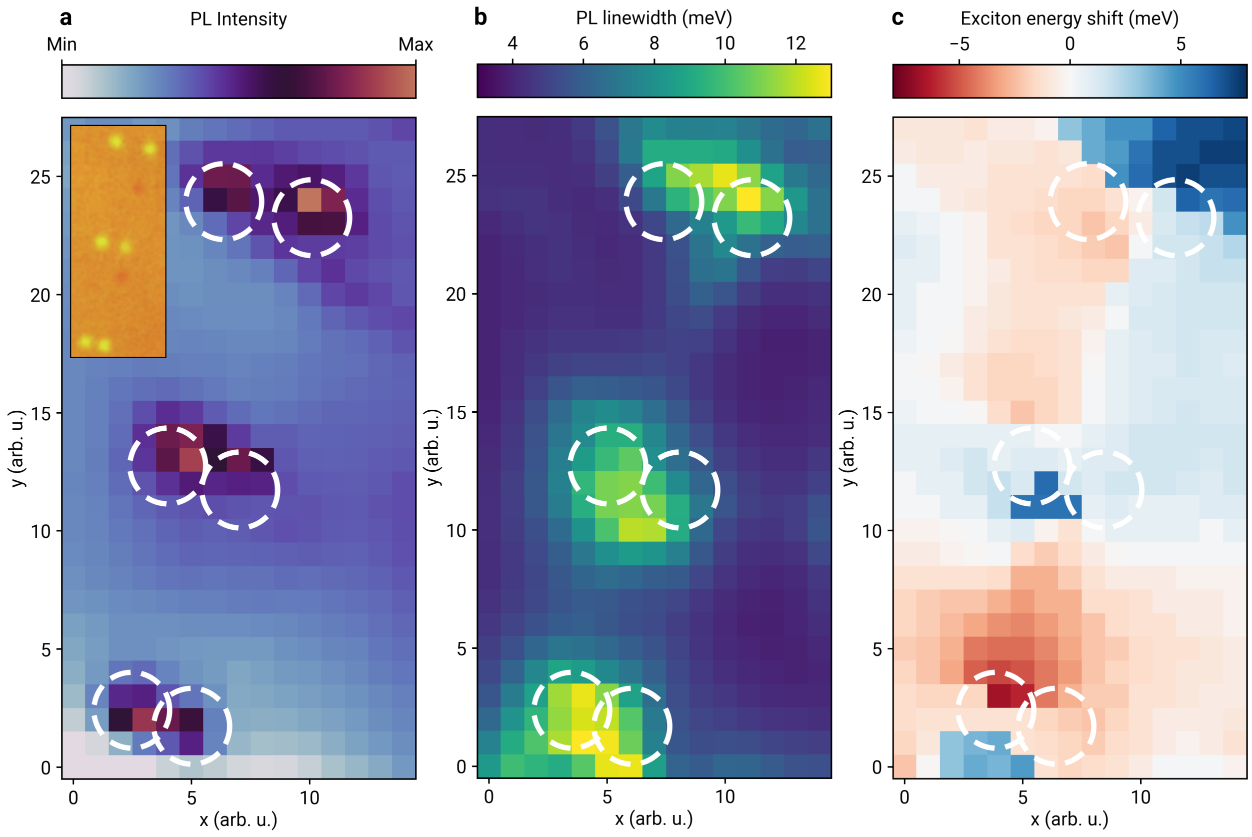

SUPPLEMENTARY FIG. 2. **Confocal photoluminescence spectroscopy.** **a**, Large-area raster-scan map of cryogenic confocal PL intensity, integrated in the spectral range 1.61 to 1.72 eV. The investigated device area, shown in the inset, contains the three etch site areas shown in Fig. 1b of the main text. **b** and **c**, PL FWHM linewidth and energy shift from the mean exciton energy, respectively, for the same sample area as in **a**, obtained by fitting a Lorentzian to the exciton PL spectra. The dashed circles indicate the point spread function of the etch sites.

change in dielectric environment [1–5]. The resonance originating from the fully encapsulated domain, at maximum PL intensity in Supplementary Fig. 1b, exhibits a pronounced blueshift at the etch site position, which likely originates from local strain [6] induced during fabrication. This blueshift is irrelevant to any claims made in the main text, as well as for the computation of lower polariton energies in Supplementary Note IV and cavity-mediated exciton-hopping in Supplementary Note V. We observe similar behavior for the etch site area P_3, as evident from Supplementary Figs. 1c and d.

A large-area raster scan map of integrated PL is shown in Supplementary Fig. 2a, with the etch sites again clearly visible as PL hotspots. In Supplementary Fig. 2b, we show the PL

FWHM linewidth for the same device area, obtained by fitting a Lorentzian to the exciton PL spectra. At the etch sites, the spectra exhibit spectral broadening, originating from the additional resonance of the redshifted domain-localized excitons. Potential additional inhomogeneous broadening could stem from local disorder induced during the fabrication process. Away from the etch sites, the PL linewidth is homogeneous across the investigated area.

SUPPLEMENTARY NOTE II: ANALYSIS OF CAVITY TRANSMISSION SPECTRA

To determine exciton resonance energies and light-matter coupling strengths from cavity-based measurements, we evoke a dissipative model for the cavity transmission. The starting point is the time-independent Hamiltonian of the system, which describes excitons of different energies E_i localized to non-overlapping areas of the device labeled i as individual quantum wells coupled to a single cavity mode, each with a light-matter coupling strength g_i,

$$H = E_C a^\dagger a + \sum_i E_i b_i^\dagger b_i + \hbar g_i (b_i^\dagger a + a^\dagger b_i). \tag{1}$$

In this expression, b_i is the bosonic annihilation operator of excitons in area i, while a is the bosonic annihilation operator for cavity photons at energy E_C. Combining Eq. 1 with the dissipative input-output formalism of Ref. [7], we find the cavity transmission at energy E

$$T(E) = \eta \kappa_\mathrm{m}^2 \left| \mathrm{i}(E_C - E) + \hbar\kappa/2 + \sum_i \frac{(\hbar g_i)^2}{\mathrm{i}(E_i - E) + \hbar\Gamma_i/2} \right|^{-2}, \tag{2}$$

in which κ and Γ_i are the cavity and exciton FWHM linewidths, respectively, and η is a proportionality constant. $\kappa_\mathrm{m} = \kappa_\mathrm{m,in}\kappa_\mathrm{m,out}$ is the product of decay rates through the in- and outcoupling mirror, $\kappa_\mathrm{m,in}$ and $\kappa_\mathrm{m,out}$, respectively. As explained in the Methods section, the linewidth of our cavity is broadened by vibrational fluctuations. By contrast, the light-matter coupling dynamics are dominated by the Lorentzian contribution to the cavity linewidth as the frequency of the mechanical vibrations is orders of magnitude slower than the energy exchange rate between the cavity and excitons. In practice, we found this distinction to have negligible impact on the data analysis procedure presented in the following, such that vibration broadening is implicitly accounted for in the value for κ in

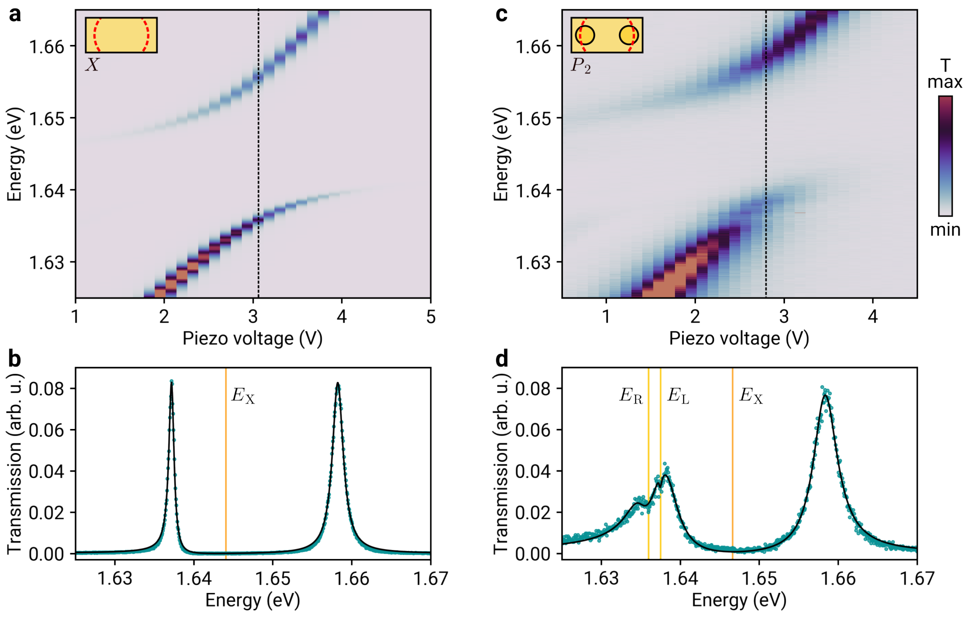

SUPPLEMENTARY FIG. 3. **Analysis of cavity transmission spectra.** **a**, Cavity transmission as function of cavity length, tuned via piezoelectric actuator voltage. The cavity mode is positioned on a TMD area fully encapsulated by hBN. **b**, Experimental transmission spectrum obtained for the voltage marked by the vertical dashed line in **a** (green dots), along with a fit of the model of Eq. 2 for a single cavity-coupled exciton resonance at energy E_X (solid line). **c**, Cavity length sweep with the cavity positioned at the etch site pair P_2, resulting in additional cavity-coupled excitonic resonances originating from the etch site domains. **d**, Same as **b**, but with a model fit for three cavity-coupled exciton resonances of different energies and coupling strengths. Resonance energies of excitons localized to the etch site domains are labeled E_L and E_R, E_X is the energy of excitons in the hBN encapsulated device area. All data in the figure are normalized to the maximum transmission measured in the respective cavity length sweep.

Eq. 2. A trion resonance with an energy near 1.62 eV coupled weakly to the cavity is irrelevant for the findings presented in this manuscript due to sizable energy detuning.

In the experiment, we measure cavity transmission as a function of cavity length, with a typical result shown in Supplementary Fig. 3a for the cavity mode positioned on the fully encapsulated TMD area. All spectra are background-corrected for a constant CCD offset and normalized with respect to the maximum measured transmission for a given length sweep. From transmission measurements of the empty cavity (covering only two

hBN layers), we found that κ_{m} varied by about 20% in the investigated spectral range, a result of wavelength-dependent mirror reflectivity. To account for this effect, we normalized the measured transmission spectra $T_{\mathrm{meas}}(E)$ by the values $\kappa^2_{\mathrm{m,meas}}(E)$ measured for the empty cavity, which we found to improve the results of the fit procedure described in the following.

To determine E_i and g_i, we fit Eq. 2 to normalized transmission spectra at different cavity lengths. Data and a representative fit result are shown in Supplementary Fig. 3b by green dots and the black line, respectively. The fit yields good agreement between model and data, also in the case of multiple exciton domains coupled to the cavity, for which representative data and fit results are shown in Supplementary Figs. 3c and d, respectively.

For each excitonic resonance i (identified by the presence of corresponding polariton branches), we compute mean values $\bar{E}_i$, $\bar{g}_i$ and standard error δE_i, δg_i from fit results for E_i and g_i obtained at different cavity lengths. $\bar{E}_i \pm \delta E_i$ and $\bar{g}_i \pm \delta g_i$ constitute our measurement results and uncertainty. To obtain good fit quality, we ensured that the fit results obtained for individual cavity lengths complied with three criteria: First, the fit results for the cavity energy should be a linear function of cavity length. Fits for which the obtained cavity energy deviated strongly from this linear dependence were discarded in the analysis. Second, only fits in which the cavity energy was in or near resonance with the exciton energy were used, a condition which we found to minimize the errors in the fit parameters of interest. Third, fits whose cost functions deviated largely from those obtained for similar cavity lengths were discarded. Fit results for different cavity lengths were used to compute $\bar{E}_i \pm \delta E_i$ and $\bar{g}_i \pm \delta g_i$, with the precise number of spectra available for analysis determined by the signal to noise ratio in the respective polariton branches.

Due to an ellipticity in the fiber mirror profile, the cavity exhibits non-degenerate higher order Hermite-Gaussian modes [8], which contribute to the measured transmission spectra. We found this contribution to depend on the position of the cavity mode relative to the etch sites, a finding we illustrate in Supplementary Fig. 4. If the center of the cavity mode was placed near the bottom of the etch site center (coordinate system defined in the inset of Supplementary Fig. 4a), the transmission spectra were typically dominated by a single cavity resonance associated with the fundamental Gaussian cavity mode, as illustrated by the data shown in Supplementary Fig. 4a. Results of the theoretical model of Eq. 2, plotted in Supplementary Fig. 4b, yield excellent agreement with the data.

If the cavity mode was moved close to the etch site center, with representative data shown

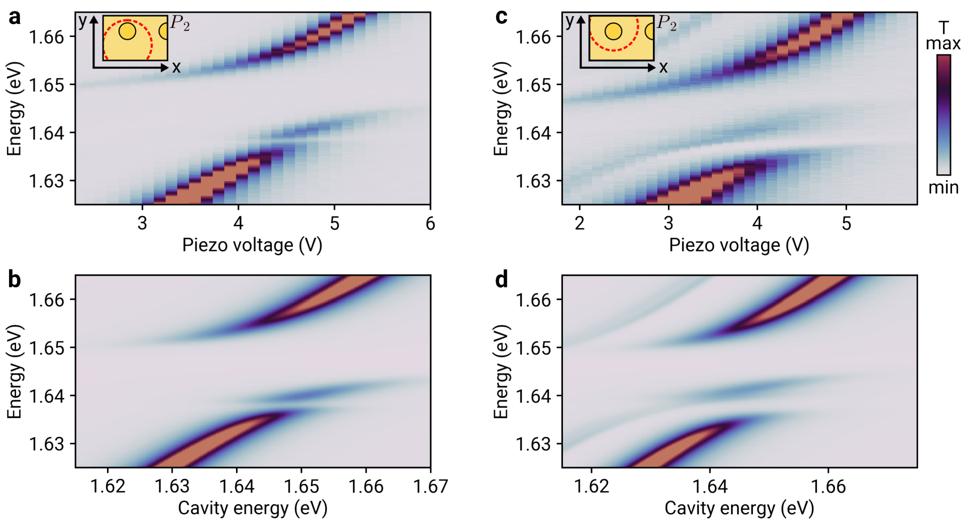

SUPPLEMENTARY FIG. 4. **Higher order transverse cavity modes.** **a**, Measured cavity transmission for a sweep of the cavity length. The position of the cavity mode with respect to the etch site pair P_2 is illustrated in the inset. Excitons in the left etch site and the encapsulated monolayer couple to the cavity, resulting in three polariton branches. **b**, Plot of theoretical cavity transmission (Eq. 2), with parameters obtained from a fit to the data in in **a**. **c**, Same as in **a**, with a different cavity mode position as illustrated in the inset. Higher order transverse cavity modes contribute additional cavity resonances to the spectra. **d**, Plot of theoretical cavity transmission, with the model of Eq. 2 extended to the case of three cavity modes coupling to the excitonic resonances. The model parameters were adjusted to yield agreement with the data in **c**.

in Supplementary Fig. 4c, higher order modes contributed cavity resonances to the measured spectra, blueshifted with respect to the fundamental mode. To support this assignment, we extend the model of Eq. 2 to describe exciton-coupling to three different cavity modes. The result is shown in Supplementary Fig. 4d and agrees well with the measurement result.

The contribution of the higher order modes to the measured spectra persisted as the cavity mode was moved towards the top of the etch site center. This asymmetry, which was also observed for the etch site pairs P_1 and P_3, is likely a result of asymmetric transversal mode profiles caused by a tilted cavity fiber. For several positions close to the etch site centers, the higher order modes were resonant with the middle polariton branches, adding uncertainty to the fit procedure described above. To determine exciton coupling strengths and resonance energies, as well as to visualize cavity-mediated exciton hopping in Figs. 3

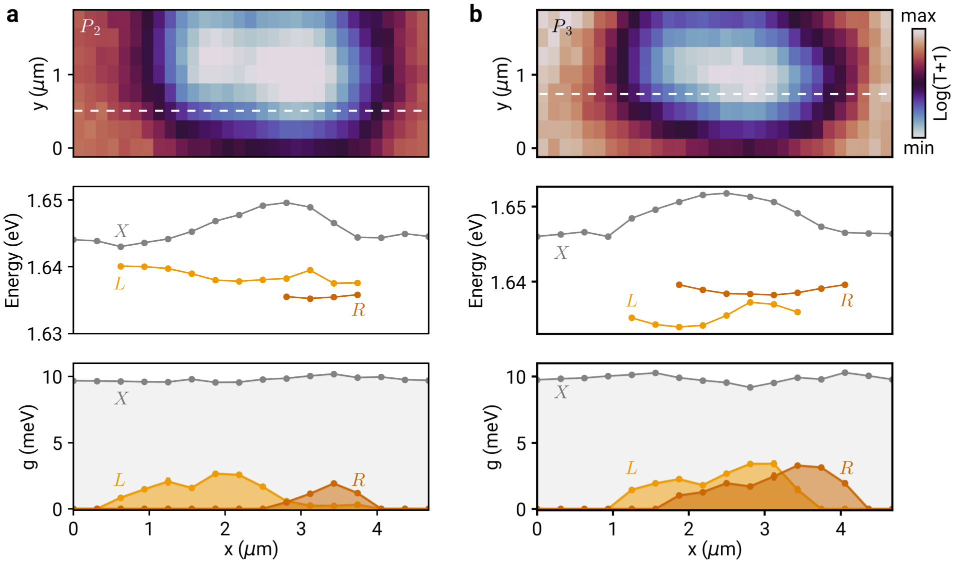

SUPPLEMENTARY FIG. 5. **Cavity-coupled exciton domains.** **a**, Top panel: Cavity transmission map at fixed cavity energy ($E_C = 1.612$ eV) of the etch site pair P_2. Axes of the coordinate system are the same as in Fig. 1b of the main text. Middle panel: Exciton resonance energies along the white dashed line in the top panel, identified from cavity length sweeps and originating from X, L and R domains as defined in the main text. Bottom panel: Exciton light matter coupling strengths g for the resonances identified in the middle panel. At positions where polariton branches associated with the individual resonances were absent in the transmission spectra, no data points are shown for the energies, and the values of g are set to zero. **b**, Same as **a** but for the etch site pair P_3. The cavity transmission map was obtained at $E_C = 1.666$ eV.

and 4 of the main text, we therefore restricted our measurements to cavity mode positions with negligible higher order mode contributions, as indicated in the insets of the respective figure panels. In the measurements of local polariton energy shifts as shown in Fig. 2 of the main text, higher order modes brightened the middle polariton branches, an effect which is negligible for the analysis in Supplementary Note IV.

SUPPLEMENTARY NOTE III: CAVITY-COUPLED EXCITON DOMAINS

To investigate the properties of dielectrically engineered exciton domains, we harnessed the tunability of our open cavity system. The top panel in Supplementary Fig. 5a shows a

raster-scan map of cavity transmission for the etch site pair P_2, obtained at a fixed cavity energy spectrally detuned from any exciton resonances. At this energy, the etch site induces mainly photonic scattering loss to the cavity mode, resulting in the observed decrease in cavity transmission.

We performed cavity length sweeps at each position along the dashed line in the transmission map of Supplementary Fig. 5a. Using the analysis procedure described in Supplementary Note II, we determined resonance energy E_i and light-matter coupling strength g_i of all exciton resonances i which contributed polariton branches to the transmission spectra. The results for E_i and g_i are shown in the middle and bottom panels of Supplementary Fig. 5a, respectively. Results for E and g at each position were labeled according to the corresponding exciton resonances X, L and R as shown in the figure.

As the cavity is placed at the left side of the etch site ($x = 0$ μm, left side in Supplementary Fig. 5a), we find a single exciton resonance labeled X with light-matter coupling strength $g_X = 9.7$ meV, which stems from the TMD area fully encapsulated with hBN. Its resonance energy experiences a blueshift as the cavity mode is moved towards the etch site centers, which is consistent with data obtained in confocal PL and likely a result of strain, as discussed in Supplementary Note I.

As expected, we find two exciton domains defined by the through holes, giving rise to resonances L and R with maximum values of light-matter coupling strength at the respective etch site centers. The maximum coupling strength $g_{L/R}$ for these domains is expected to scale as $g_{L/R} \propto \sqrt{\eta_A}$ [9, 10] with η_A the overlap between exciton domain and cavity mode. Using the domain area A_S and the $1/e^2$ area of the transverse cavity field A_C, we estimate $\max(g_{XL})\sqrt{A_S/A_C} = 2.91$ meV, close to the maximum measured value $g_L = 2.65 \pm 0.04$ meV in Supplementary Fig. 5b. The difference in energy and coupling strengths between the L and R domains reflect typical inhomogeneities in TMD-based van der Waals heterostructures. On the length scale of the cavity mode waist, the spatial variations in exciton energy are on the order of the typical exciton linewidth for all three resonances in Supplementary Fig. 4. As a result, these variations will add to the inhomogeneous broadening of the exciton linewidth. In our analysis, we therefore treat each exciton in Supplementary Fig. 4 as an inhomogeneously broadened resonance with a single, spatially dependent frequency.

Repeating measurements and analysis for the etch site pair P_3, with data shown in Supplementary Fig. 5c, yields similar results as for P_2. Again, we find different excitonic domains

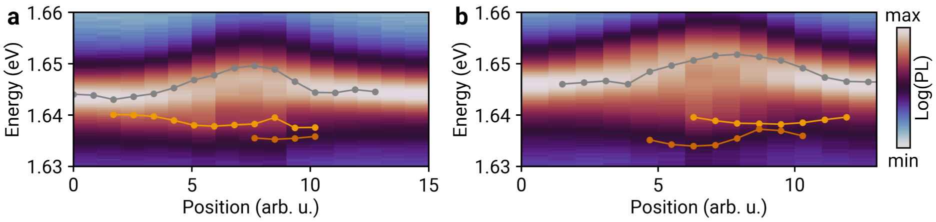

SUPPLEMENTARY FIG. 6. **Exciton resonance energies.** **a**, Confocal PL linecut for P_2, measured along the dashed line in Supplementary Fig. 1a, along with exciton resonance energies determined from the analysis of the cavity transmission spectra as shown in Supplementary Fig. 4a (colored dots, position axis scaled and shifted to match the PL emission profile). **b**, Same as **a** but for the etch site pair P_3.

L and R defined by the through-holes, whose coupling strength maxima are observed at smaller distance than for P_2 due to the reduced etch site distance. In Supplementary Fig. 6, we show the resonance energies obtained by analyzing the cavity transmission spectra along with confocal PL linecuts. The exciton energies obtained from cavity transmission correspond to the spectral position of the dominant confocal PL resonance and its redshifted shoulder.

Our observation of redshifted exciton resonaces at reduced dielectric screening is in agreement with recent results [1, 5]. Notably, it contrasts the case of graphene-encapsulated TMD monolayers [2], for which the exciton energy is known to blueshift at reduced dielectric screening, which is a result of the difference in dielectric response of graphene and hBN [5]. For the sake of completeness, we note that we observed spectrally broad, blueshifted exciton resonances of unknown origin for a small number of etch sites, which coupled weakly to the cavity and are irrelevant to the measurements presented in the following. We summarize that our fabrication method allows to deterministically create exction domains redshifted in resonance energy by up to 10 meV from excitons in the hBN encapsulated monolayer area, with coupling strengths of approx. 2.5 meV defined by the domain size.

SUPPLEMENTARY NOTE IV: LOCAL POLARITON ENERGY SHIFTS

In Fig. 2 of the main text, we demonstrate local polariton energy shifts at the etch site positions. To visualize these shifts, we plot the transmission as a function of cavity mode

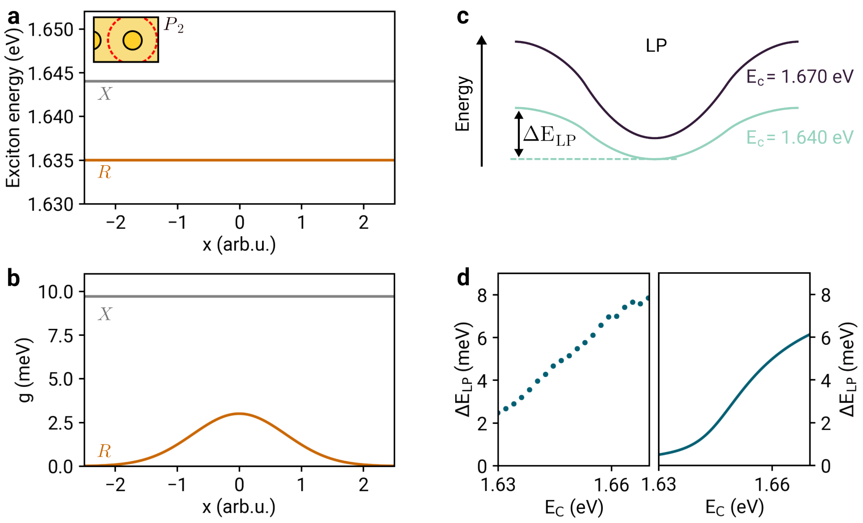

SUPPLEMENTARY FIG. 7. **Model for local polariton energy shifts.** **a**, **b**, Exciton energy and light-matter coupling strength, respectively, used to model the polariton energy shifts for the right etch site of P_2 illustrated in the inset. X (R) resonances stem from excitons localized to the encapsulated monolayer (right etch site domain), as demonstrated in Supplementary Note III. **c**, Schematic of the lower polariton (LP) energy shift at a single exciton domain for different cavity energies, as observed in experiments presented in the main text. **d**, LP energy shift ΔE_{LP} for the etch site R of P_2 as obtained from experiment (left panel) and calculations as outlined in Supplementary Note IV (right panel).

position, measured at constant cavity energy E_C. E_C was determined from a fit of the dissipative model of Supplementary Note II at a position away from any etch site (e.g. $x = 0$ μm). At each position, the spectrum was normalized to the maximum transmission of the lower polariton branch. The polariton energies, shown as dots in Fig. 2d of the main text, were determined as the energies of maximum cavity transmission of lower and upper polariton branch for each position. Polariton energies were fit with the double Gaussian profile

$$E(x) = c - a_L \exp(-(x - x_L)^2/s_L^2) - a_R \exp(-(x - x_R)^2/s_R^2), \tag{3}$$

where the fit results for a_L and a_R are the local energy shifts at the left and right etch site, respectively, shown Fig. 2c of the main text. For the single etch site in Fig. 2a of the main text, we modified Eq. 3 to describe a single Gaussian well with the offset c a linear function of position.

To elucidate the mechanism of the local energy shift, we focus on the right etch site of P_2, as illustrated in the inset of Supplementary Fig. 7a. Energy and light-matter coupling strength of the relevant exciton resonances are shown in Supplementary Figs. 7a and b, respectively. The spatial dependence is based on the measurements presented in Supplementary Fig. 5b, with the coupling strength for R excitons approximated by a Gaussian profile with the etch site center positioned at $x = 0$ for illustration purposes. The blueshift in X energy observed in Supplementary Fig. 7 is negligible for the computations presented in the following.

As evident from Fig. 2 of the main text, we observe an energy shift of magnitude ΔE_{LP} for the lower polariton branch, which increases with increasing cavity energy, as illustrated in Supplementary Fig. 7c. As explained in the main text, this observation results from the fact that as the cavity energy is increased, the lower polariton energy approaches that of the energetically lowest cavity-coupled exciton. As a result, ΔE_{LP} monotonously increases until it reaches $E_X - E_R$.

To provide additional verification, we compute the energy shift as $\Delta E_{\mathrm{LP}} = E_{LP}(x = \infty) - E_{LP}(x = 0)$, with the lower polariton energy $E_{LP}(x)$ obtained from the eigenstates of Eq. 1 for the values of E and g shown in Supplementary Figs. 7a and b. The result, shown in the right panel of Supplementary Fig. 7d, is in good agreement with the data obtained for P_2, which is reproduced in the left panel of the figure.

SUPPLEMENTARY NOTE V: REGIME OF DISPERSIVE CAVITY COUPLING

To describe our system in the regime of dispersive cavity coupling, i.e. for large detunings between cavity energy and all exciton resonances, we transform and expand the time-independent, non-dissipative Hamiltonian of Eq. 1 as described in the main text. We note that the resulting effective coupling of strength J_{ij} has been studied for two or multiple individual (fermionic) two-level systems coupled to the same cavity mode, such as superconducting qubits [11] and defects in diamond [7]. This contrasts the case of bosonic excitons studied in our work.

To obtain the values for the cavity-mediated exciton hopping J_{XL} shown in Fig. 3c of the main text from sweeps of the cavity length as shown in Fig. 3b, we identified zeros in the derivative of the cavity transmission as resonance energies. We then fitted the energy $E_{\mathrm{S1/S2}}$

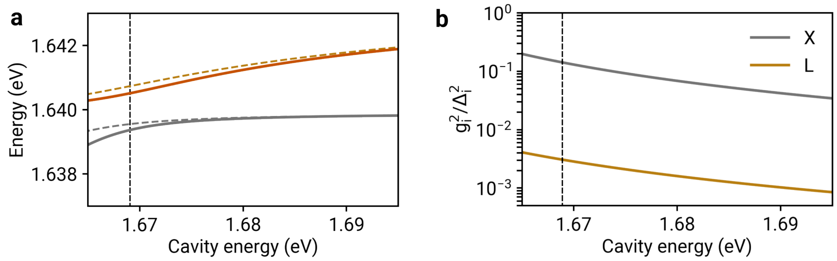

SUPPLEMENTARY FIG. 8. **Regime of dispersive cavity coupling.** **a**, Eigenstates of the effective Hamiltonian for the dispersive regime of cavity coupling (Eq. 1 of the main text, solid lines) and of the full polariton Hamiltonian of Supplementary Eq. 1 (dashed lines). The parameters used for the computation correspond to the experimental setting in Fig. 3 of the main text and are given in Supplementary Note V. In the framework of dispersive cavity coupling, the gray (orange) branch is predominantly X-(L-)exciton-like. **b**, $|g_i^2/\Delta_i|^2$, computed for the two exciton domains X and L. The dashed vertical line in both panels indicates the cavity energy at which the experimental branches exhibited minimum splitting J_{XL}.

of the two branches with a model for two coupled oscillators, $E_{\mathrm{S1/S2}} = (E_{\mathrm{X1}} + E_{\mathrm{X2}})/2 \pm \sqrt{J_{\mathrm{XL}}^2 + (E_{\mathrm{X1}} - E_{\mathrm{X2}})^2/2}$, with E_{X1} a constant and E_{X2} a linear function of the cavity length, to obtain J_{XL}. The theoretical eigenstates of the effective Hamiltonian shown in Fig. 3b of the main text were computed for two cavity-coupled exciton resonances X and L using the following set of parameters: $E_X = 1.6436$ eV, $g_X = 9.5$ meV, $E_L = 1.6399$ eV, $g_L = 1.6$ meV. These values are in excellent agreement with the results obtained from fits of Eq. 2 to the transmission spectra for different cavity lengths measured at the same cavity mode position: $E_X = 1.64358 \pm 0.00002$ eV, $g_X = 9.577 \pm 0.004$ meV, $E_L = 1.63999 \pm 0.00003$ eV, $g_L = 1.5 \pm 0.1$ meV. From the same fits, we obtained the cavity energy as a function of piezoelectric actuator voltage used in the theoretical computation.

The strict theoretical requirement for Eq. 1 of the main text to hold is $|g_i^2/\Delta_i|^2 \ll 1$ for each excitonic species. In practice, we verified by numerical analysis that the deviation between the dissipative model and the true eigenstates of the full Hamiltonian of Eq. 1 depended on the precise values of coupling strengths and energies of the individual exciton resonances. For the data shown in Fig. 3 of the main text, two exciton resonances differing in energy by approximately 3 meV and with values of g_i differing by a factor of approximately 6 coupled to the cavity with detunings $\Delta_X \approx 15$ meV. For these parameters, we found

that deviations between polariton branches as predicted by Eq. 1 of the main text and the true eigenstates of the system were well within the typical linewidths observed in the experiments. This finding is illustrated in more detail in Supplementary Fig. 8a, which shows the eigenstates of both the full polariton Hamiltonian of Supplementary Eq. 1 (dashed lines) as well as for the dispersive Hamiltonian Eq. 1 of the main text (solid lines). The computation was performed using the parameters for the theoretical prediction shown in Fig. 3b. The agreement between the two models underlines the validity of the approximation even at cavity lengths where $|g_i^2/\Delta_i|^2 \ll 1$ does not strictly hold (with $|g_i^2/\Delta_i|^2 < 0.1$ in Supplementary Fig. 8b). We note that this regime of dispersive cavity-detuning could only be accessed at selected positions on the sample. For different positions, broadening and reduced transmission of the polariton branches induced by disorder resulted in reduced signal-to-noise ratio, preventing the extraction of the system's eigenstates at satisfactory experimental confidence.

The theoretical transmission profile shown in Fig. 4b of the main text was obtained from a fit of Eq. 2, yielding the parameters: $E_X = 1.6431 \pm 0.0002$ eV, $g_X = 9.5322 \pm 0.0001$ meV, $E_L = 1.63750 \pm 0.00001$ eV, $g_L = 0.4 \pm 0.4$ meV, $E_R = 1.63582 \pm 0.00005$ eV, $g_R = 1.2 \pm 0.4$ meV. In Supplementary Fig. 9a, we show a cavity length sweep obtained at the same position. The dashed black lines show the evolution of the branches computed according to Eq. 1, with the exciton parameters $E_X = 1.643$ eV, $g_X = 8.5$ meV, $E_L = 1.6377$ eV, $g_L = 0.6$ meV, $E_R = 1.636$ eV, $g_R = 1.15$ meV, close to the values obtained from the dissipative model fit at the respective position. For cavity energies above 1.651 eV, the model of Eq. 1 is in good agreement with the experimental observations, placing our system in the dispersive cavity-coupling regime. Operation in this regime is confirmed by small values of $|g_i^2/\Delta_i|^2$ shown in Supplementary Fig. 9b, similar to Supplementary Fig. 8b discussed above. The values for J_{LR} shown in Fig. 4c were computed from the measured values of $E_{L/R}$ and $g_{L/R}$ shown in Supplementary Fig. 4 for a cavity energy of 1.670 eV.

SUPPLEMENTARY NOTE VI: CAVITY CHARACTERISTICS

To characterize our cavity system, we determined the longitudinal mode order from transmission spectra of the empty cavity. Along the optical axis, the mirror distance can be coarse-tuned over the range of a few mm via a piezoelectric nanopositioner, while fine-

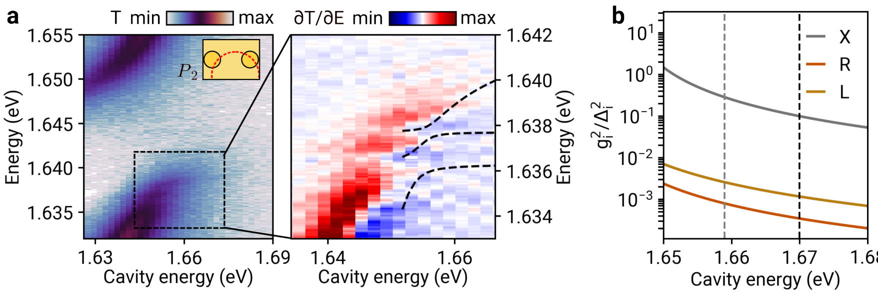

SUPPLEMENTARY FIG. 9. **Dispersive cavity regime for two coupled exciton disks. a**, Left panel: cavity transmission as a function of cavity energy, with the cavity mode covering domains L and R as illustrated in the inset. Right panel: derivative of cavity transmission with respect to energy, computed for data in the dashed rectangle in the left panel. The dashed lines are the eigenstates of the effective system Hamiltonian, Eq. 1 of the main text, computed for the parameters given in Supplementary Note V. The data are taken at the same position as the spectrum in Fig. 4b of the main text. **b**, $|g_i^2/\Delta_i|^2$, computed for the three exciton resonances X, L and R visible in **a**. The black dashed line indicates the cavity energy at which the values for J_{XL} in Fig. 4c of the main text were obtained. The gray dashed line indicates the cavity energy for the transmission spectrum shown in Fig. 4b of the main text.

tuning within a smaller range is achieved with an additional piezoelectric transducer. To determine the mode order, we tuned the mirror distance such that two fundamental transverse Gaussian modes of neighboring longitudinal moder order were visible in the spectrum. A representative transmission measurement is shown in Supplementary Fig. 10a. From the wavelengths λ_1 and λ_2 of these two resonances, assigned such that $\lambda_2 > \lambda_1$, we computed the effective cavity length as $L_{\mathrm{eff}} = \lambda_1\lambda_2/2/(\lambda_2 - \lambda_1)$. We note that this effective length accounts for the penetration depth of the field in the DBR mirrors. The mode order for a given resonance wavelength λ_i was calculated from $L_{\mathrm{eff}} = q\lambda_i/2$.

Using the piezoelectric nanopositioner, we reduced the mirror distance to minimize the mode order and, correspondingly, the cavity mode volume. Supplementary Fig. 10b shows a transmission spectrum with the cavity length reduced compared to Fig. 10a, such that the mode order $q = 10$ now contributes a peak to the spectrum. The increased free spectral range (i.e. the distance between the resonance peaks) compared to the measurement shown in Fig. 10a directly indicates a reduced mode order in this measurement. For mode orders

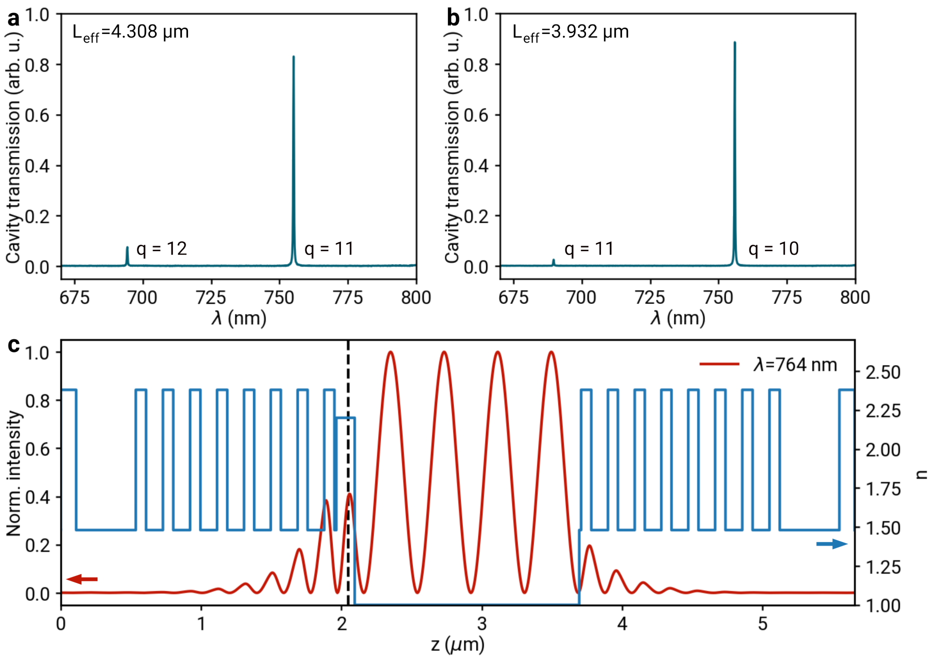

SUPPLEMENTARY FIG. 10. **Fiber cavity characteristics.** **a**, Experimental transmission spectrum of the empty fiber cavity. The effective cavity length $L_{\text{eff}} = 4.308$ μm was determined as described in the main text. The two peaks correspond to the resonances of the fundamental transverse modes with different effective longitudinal mode order q. **b**, Same as **a** but for $L_{\text{eff}} = 3.932$ μm. **c**, Intensity of the intracavity light field along the optical axis from a transfer matrix calculation (solid red line). The assumed mirror distance is 1.600 μm, yielding a resonance wavelength of 764 nm. The refractive index profile along the structure is shown in blue. The vertical dashed line indicates the position of the TMD monolayer, encapsulated between two hBN layers.

$q < 9$, the free spectral range became so large that modes with larger q had resonance wavelengths outside the mirror stopband and could thus not be detected in the experiment. For mirror distances below this mode order, we therefore minimized the cavity length in small steps, which allowed us to keep track of the mode order, until reaching the smallest order $q = 6$ for which the mirrors were not in contact.

The simulated intensity distribution of the intra-cavity light field along the optical axis is shown in Supplementary Fig. 10c, together with the refractive index profile of the full cavity. The chosen mirror distance of 1.600 μm results in five antinodes between the mirrors, which together with the penetration into the DBR coatings is estimated to correspond to a mode

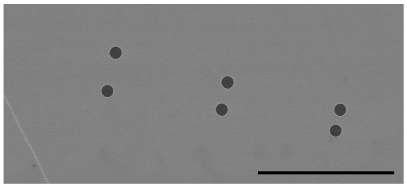

SUPPLEMENTARY FIG. 11. **Processing of hexagonal boron nitride.** SEM image of a processed hBN flake, showing pairs of circular through-holes also visible in Fig. 1b of the main text. An identical pattern was used for the flake in the fabricated device, which was not imaged in SEM to avoid contamination caused by carbon deposition. The scale bar is 10 μm.

order $q = 6$. The black dashed vertical line indicates the position of the TMD monolayer, which is encapsulated by hBN and placed close to an antinode of the cavity field at maximum light-matter coupling strength.

SUPPLEMENTARY REFERENCES

[1] Khestanova, E., Shahnazaryan, V., Kozin, V. K., Kondratyev, V. I., Krizhanovskii, D. N., Skolnick, M. S., Shelykh, I. A., Iorsh, I. V. & Kravtsov, V. Electrostatic control of nonlinear photonic-crystal polaritons in a monolayer semiconductor. *Nano Letters* **24**, 7350–7357 (2024).

[2] Raja, A., Chaves, A., Yu, J., Arefe, G., Hill, H. M., Rigosi, A. F., Berkelbach, T. C., Nagler, P., Schüller, C., Korn, T., Nuckolls, C., Hone, J., Brus, L. E., Heinz, T. F., Reichman, D. R. & Chernikov, A. Coulomb engineering of the bandgap and excitons in two-dimensional materials. *Nature Communications* **8**, 15251 (2017).

[3] Peimyoo, N., Wu, H.-Y., Escolar, J., De Sanctis, A., Prando, G., Vollmer, F., Withers, F., Riis-Jensen, A. C., Craciun, M. F., Thygesen, K. S. & Russo, S. Engineering Dielectric Screening for Potential-well Arrays of Excitons in 2D Materials. *ACS Applied Materials & Interfaces* **12**, 55134–55140 (2020).

[4] Borghardt, S., Tu, J.-S., Winkler, F., Schubert, J., Zander, W., Leosson, K. & Kardynał, B. E. Engineering of optical and electronic band gaps in transition metal dichalcogenide monolayers through external dielectric screening. *Physical Review Materials* **1**, 054001 (2017).

[5] Ben Mhenni, A., Van Tuan, D., Geilen, L., Petrić, M. M., Erdi, M., Watanabe, K., Taniguchi, T., Tongay, S. A., Müller, K., Wilson, N. P., Finley, J. J., Dery, H. & Barbone, M. Breakdown

of the Static Dielectric Screening Approximation of Coulomb Interactions in Atomically Thin Semiconductors. *ACS Nano* **19**, 4269–4278 (2025).

[6] Schmidt, R., Niehues, I., Schneider, R., Drüppel, M., Deilmann, T., Rohlfing, M., de Vasconcellos, S. M., Castellanos-Gomez, A. & Bratschitsch, R. Reversible uniaxial strain tuning in atomically thin WSe_2. *2D Materials* **3**, 021011 (2016).

[7] Evans, R. E., Bhaskar, M. K., Sukachev, D. D., Nguyen, C. T., Sipahigil, A., Burek, M. J., Machielse, B., Zhang, G. H., Zibrov, A. S., Bielejec, E., Park, H., Lončar, M. & Lukin, M. D. Photon-mediated interactions between quantum emitters in a diamond nanocavity. *Science* **362**, 662–665 (2018).

[8] Benedikter, J., Hümmer, T., Mader, M., Schlederer, B., Reichel, J., Hänsch, T. W. & Hunger, D. Transverse-mode coupling and diffraction loss in tunable Fabry-Pérot microcavities. *New Journal of Physics* **17**, 053051 (2015).

[9] Thureja, D. *Electrically tunable quantum confinement of neutral excitons*. Doctoral Thesis, ETH Zurich (2023).

[10] Gebhardt, C., Förg, M., Yamaguchi, H., Bilgin, I., Mohite, A. D., Gies, C., Florian, M., Hartmann, M., Hänsch, T. W., Högele, A. & Hunger, D. Polariton hyperspectral imaging of two-dimensional semiconductor crystals. *Scientific Reports* **9**, 13756 (2019).

[11] Majer, J., Chow, J. M., Gambetta, J. M., Koch, J., Johnson, B. R., Schreier, J. A., Frunzio, L., Schuster, D. I., Houck, A. A., Wallraff, A., Blais, A., Devoret, M. H., Girvin, S. M. & Schoelkopf, R. J. Coupling superconducting qubits via a cavity bus. *Nature* **449**, 443–447 (2007).
